# Editorial: Microbiome in the growth and management of different types of cancer

**DOI:** 10.3389/fmolb.2023.1249301

**Published:** 2023-07-18

**Authors:** Abdul Arif Khan, Dietrich Büsselberg, Gislane Lelis Vilela de Oliveira, Shahanavaj Khan

**Affiliations:** ^1^ Division of Microbiology, ICMR-National AIDS Research Institute (ICMR), Pune, India; ^2^ Weill Cornell Medicine- Qatar, Ar-Rayyan, Qatar; ^3^ Laboratory of Immunomodulation and Microbiota, Department of Chemical and Biological Sciences, Institute of Biosciences (IBB), Sao Paulo State University (UNESP), Botucatu, Brazil; ^4^ Department of Medical Lab Technology, Indian Institute of Health and Technology (IIHT), Deoband, India

**Keywords:** microbiome, cancer, growth, diagnosis, treatment

Despite multiple attempts to understand its molecular mechanisms, cancer continues to pose a significant challenge to both the general population and healthcare workers. Multiple studies have highlighted the important role of microorganisms in almost all aspects of cancer (Khan et al., 2012; Shahanavaj et al., 2015). The fate of any cell, especially the growth and development of cancer cells, is regulated by a number of cellular interactions, including host and pathogen interaction during the microbial contribution in carcinogenesis. The current Research Topic tries to gather the role of the microbiome in the process of carcinogenesis. All the articles compiled in the current Research Topic provide in-depth coverage of the current research in the field and their relevance with the objectives of compiling this Research Topic.

One of the most common and deadliest types of cancer is colorectal cancer (CRC). Yi-Hui and George show the important aspects for the prediction of colorectal cancer (CRC) using 16S rRNA or shotgun metagenomics data through a specific algorithms approach. The approach presented in the article demonstrates the key CRC-related taxa including predominant levels of *Bacteroides fragilis* (*B. fragilis*). The importance of identifying specific microbiota constituents such as of *B. fragilis* may be helpful to reveal the microbial signatures through molecular techniques which may provide valuable information for the disease pathology. In addition, the outcome of the study will open avenues for the management of CRC.

DNA methyltransferase (DNMT) is an important enzyme which controls the process of DNA methylation. Narabayashi et al. explore the involvement of the adaptor molecule RBM4 in the recruitment of DNMT. The study also shows the role of gut microbiota in the TLR4 gene methylation in the colonic epithelial cells (CEC) line through increasing RBM14 expression. RBM14 is an adaptor molecule controlling the recruitment of DNMT that bind to specific target genes. Furthermore, compared to cells from germ-free mice, the results of this study report the overexpression of RBM14 in conventional mice colonic epithelial cells. Collectively, the results show that the gut microbiota mediates TLR4 methylation in colonic epithelial cells through RBM14 upregulation. These results indicate that the intestinal microbiota helps in intestinal homeostasis through epigenetic regulation, corroborating recently published work (Lee et al., 2022). 

The article by Sarfraz et al. discusses the very important aspect of metabolic regulation by nutrition and microbiota. Metabolic regulation by microbiota and nutrition is an important aspect for physiological regulation in multiple diseases including cancer. This article discusses the disparities among microbiota composition and provides the relationship between diet and microbiota with a focus on using this association to harness microbiota modulation through diet for physiological benefits. The article demonstrates various host and exogenous factors which alter the composition of gut microbiota. The article also emphasizes the role of lifestyle and diet on microbiota composition. Moreover, the work shows the important information of the gut microbiome on dietary interventions and the possible effect of nutrition including feeding pattern (fasting, dietary intake) and circadian patterns which may affect the host metabolism and resultant intestinal microbial population. Host metabolism plays an important role in the progression of cancer and therefore the information presented in the article provides a valuable addition to the field by shedding light on the metabolic regulation by microbiota through diet. The consumed polysaccharides from a diet play a crucial role in the modulation of intestinal microbiota and their functions. The microbial metabolites such as short-chain fatty acids (SCFAs) have the potential to alter the physiology of the host, and act as prominent factors to promote certain immunological disorders and metabolic disorders including cancer and obesity. Hence, personalized nutrition may be used for the management and cure of various immune diseases, metabolic disorders, neurological disorders, and cancer.

Approximate 90% of most of oral cancers belong to types of oral squamous cell carcinoma (OSCC).

Saproo et al. present a brief research report on the saliva samples of OSCC patients and normal controls. The report presents findings about the differential gene expression analysis of salivary RNAs from OSCC patients. The findings also illustrate potential salivary indicators of OSCC and their relationship to different aspects of carcinogenesis. Notably, the study also explores microbial dysbiosis among OSCC patients and their relationship with tumor-promoting pathways. Though few other studies present microbiota dysbiosis among OSCC patients, the study involving the detection of the integrated landscape for OSCC specific salivary RNA from an Indian population provides a valuable addition to the field. The study found that Prevotella is significantly enriched in OSCC and indicated that differently abundant microbial taxa are also involved in pathways associated with carcinogenesis. The role of the *Prevotella* genus in inflammation has been presented in several diseases (Larsen, 2017; Khan and Khan, 2020). The differently abundant microbial taxa provide valuable insights into understanding the whole mechanism of OSCC progression.

Overall, the articles gathered in the current research provide a valuable source for understanding the different perspectives about microbiota-mediated cancer and their implications in the development, diagnosis, and management of various cancer types. The high throughput host-microbiota interaction data is generated by current methods and gathered in several databases. The articles included in the current Research Topic create a thought-provoking space and update the knowledge status for microbiota involvement in cancer, which in turn may be helpful for their utilization in the diagnosis, therapy, and management of different types of cancer.
